# Potential antimicrobial, antidiabetic, catalytic, antioxidant and ROS/RNS inhibitory activities of *Silybum marianum* mediated biosynthesized copper oxide nanoparticles

**DOI:** 10.1039/d2ra01929a

**Published:** 2022-05-11

**Authors:** Junaid Iqbal, Anisa Andleeb, Hajra Ashraf, Bisma Meer, Azra Mehmood, Hasnain Jan, Gouhar Zaman, Muhammad Nadeem, Samantha Drouet, Hina Fazal, Nathalie Giglioli-Guivarc'h, Christophe Hano, Bilal Haider Abbasi

**Affiliations:** Department of Biotechnology, Quaid-i-Azam University Islamabad 45320 Pakistan juniqbal34@gmail.com anisaandleeb@bs.qau.edu.pk hajraashraf67@gmail.com bismameer786@gmail.com gzaman@bs.qau.edu.pk bhabbasi@qau.edu.pk; Stem Cell & Regenerative Medicine Lab, National Centre of Excellence in Molecular Biology, University of Punjab 87-West Canal Bank Road Lahore 53700 Pakistan azramehmood@cemb.edu.pk; Institute of Biochemical Sciences, National Taiwan University Taipei City 10617 Taiwan rhasnain849@gmail.com; Institute of Integrative Biosciences, CECOS University Peshawar 25100 Pakistan m.nadeem@cecos.edu.pk; Laboratoire de Biologie des Ligneux et des Grandes Cultures (LBLGC), INRAE USC1328, Université d'Orléans 45067 Orléans Cedex 2 France samantha.drouet@univ-orleans.fr hano@univ-orleans.fr; Pakistan Council of Scientific and Industrial Research (PCSIR) Laboratories Complex Peshawar 25120 Pakistan hina_fazalso@yahoo.com; EA2106 Biomolécules et Biotechnologies Végétales, Université Francois-Rabelais de Tours Tours France nathalie.guivarch@univ-tours.fr

## Abstract

Use of medicinal plants for the biosynthesis of nanoparticles offers several advantages over other synthesis approaches. Plants contain a variety of bioactive compounds that can participate in reduction and capping of nanoparticles. Plant mediated synthesis has the leverage of cost effectiveness, eco-friendly approach and sustained availability. In the current study *Silybum marianum*, a medicinally valuable plant rich in silymarin content, is used as a reducing and stabilizing agent for the fabrication of nanoparticles. Biosynthesized CuO-NPs were characterized using High Performance Liquid Chromatography (HPLC), Fourier Transform Infrared Spectroscopy (FTIR), X-ray Diffraction (XRD), Scanning Electron Microscopy (SEM), and Dynamic Light Scattering (DLS) techniques. Characterization revealed that CuO-NPs having a crystalline structure showed spherical morphology with an average size of 15 nm. HPLC analysis demonstrated conjugation of various silymarin components, especially the presence of silybin A (705.06 ± 1.59 mg g^−1^ DW). CuO-NPs exhibited strong bactericidal potency against clinically important pathogenic bacterial strains *e.g. Enterobacter aerogenes* and *Salmonella typhi* with an inhibition zone of 18 ± 1.3 mm and 17 ± 1.2 mm, respectively. Synthesized nanoparticles indicated a dose dependent cytotoxic effect against fibroblast cells exhibiting a percentage cell viability of 83.60 ± 1.505% and 55.1 ± 1.80% at 25 μg mL^−1^ and 100 μg mL^−1^ concentration, respectively. Moreover, CuO-NPs displayed higher antioxidant potential in terms of (TAC: 96.9 ± 0.26 μg AAE/mg), (TRP: 68.8 ± 0.35 μg AAE/mg), (DPPH: 55.5 ± 0.62%), (ABTS: 332.34 μM) and a significant value for (FRAP: 215.40 μM). Furthermore, enzyme inhibition assays also exhibited excellent enzyme inhibition potential against α-amylase (35.5 ± 1.54%), urease (78.4 ± 1.26%) and lipase (80.50.91%), respectively. Overall findings indicated that biosynthesized CuO-NPs possess immense *in vitro* biological and biomedical properties and could be used as a broad-spectrum agent for a wider range of biomedical applications.

## Abstract 2

Use of medicinal plants for the biosynthesis of nanoparticles offers several advantages over other synthesis approaches.

## Graphical abstract

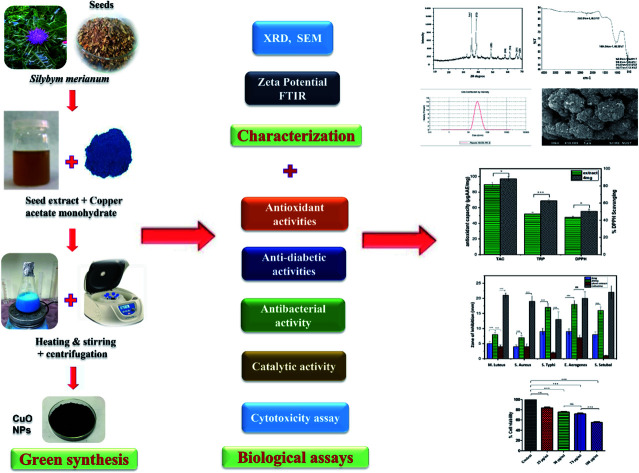

## 1. Introduction

Nanotechnology is one of the most dynamic and innovative fields of science and technology, having a multitude of applications ranging from medicines to engineering.^1–4^ Various methods such as physical, chemical and green routes have been used to fabricate these nanoscale materials, however these methods have a lot of drawbacks. From the last few decades, green approaches are becoming methods of choice for the fabrication of nanoparticles (NPs), because of their biocompatibility, safety, minimal toxicity and cost effectiveness, which makes them more ideal than other counterparts.^5,6^ Metallic nanoparticles offer a wide variety of applications mainly attributed to their small size and high surface area.^7–9^ Among other metallic nanoparticles, copper oxide nanoparticles (CuO-NPs) have been used in multiple sectors including the biomedical, textile, catalysis and sensing industries.^10–17^ Moreover, CuO is comparatively cheaper than silver, can be mixed easily with the polymers and is quite stable regarding physical and chemical properties. Different natural sources are being utilized for the production of CuO-NPs including plants, microbes, and fungi *etc.*^18–21^ Plant extract consists of variety of biomolecules and metabolites including vitamins, carbohydrates, phenolics and flavonoids, which can act as a reducing and stabling agent and can covert Cu^2+^ ions into CuO-NPs.^5,22,23^

Excessive production of free radicals in the body are key contributors of degenerative diseases including cataracts, cardiovascular diseases, cancer, brain dysfunction, and a weakened immune system.^24^ These free radicals, however, can be deactivated using antioxidants, before they attack body cells and manifest a disease. CuO-NPs are particularly renowned for effectively scavenging free radicals containing oxygen groups.^24^ CuO and other highly ionic metal oxide nanoparticles may be of significance due to their unusual crystal surface structures and high surface areas.^25^ CuO-NPs, along with their antioxidant activities, exhibit antibacterial activities against all strains of Gram-positive and -negative bacteria.^26–28^ Antibacterial activities of CuO-NPs have been reported against *Staphylococcus aureus*, *Enterococcus faecalis*, *Klebsiella pneumonia*, *Proteus vulgaris*, *Salmonella typhimurium*, *Pseudomonas aeruginosa*, *Escherichia coli*, and *Bacillus subtilis*. Among these, *E. faecalis*, and *E. coli* reported the highest activity upon treatment with CuO-NP.^29^

*Silybum marianum* belongs to the family *Asteraceae* and is enriched with a variety of bio ingredients, in particular silymarin: A major player which have been reported in treating liver^30,31^, kidney,^32^ spleen and gallbladder disorders.^33^ It was a native plant of Asia and Southern Europe however, it is now easily available across the world. *Silybum marianum* (milk thistle) is 200 cm long plant consist of conical shaped cottony stem, pale green leaves with white veins and purple–red flower. Seed extract of *Silybum marianum* contains up to 4% of the total silymarin.^34^ Aqueous extract of *Silybum marianum* consists of phenolic (silybin A, silybin B, isosilybin A, isosilybin B, silydianin, isosilychristin, silychristin) and flavonoid (taxifolin) which can serve as reducing and capping agent to enhance the biomedical properties of CuO-NPs. Green synthesis of CuO-NPs with number of biomedical importance has been reported previously using different plant parts such as Hull extract of Oak,^35^ dry black beans,^36^ peel extract of *Punica granatum*^37^ and leaf extract of aloe vera.^38^

**Fig. 1:**
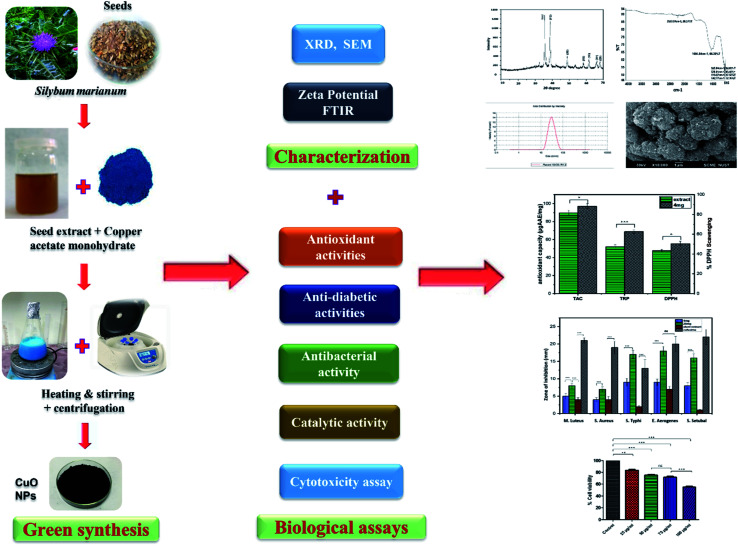
Schematic diagram of green synthesis, characterization and biological applications of CuO-NPs.

The current study is aimed to use seed extract of *Silybum marianum* as a reducing/capping agent for biosynthesis of CuO-NPs and to investigate the antibacterial and other biomedical potential of biosynthesized CuO-NPs (Fig. 1). To the best of our knowledge, this is the first report on *Silybum marianum* mediated biosynthesis of copper oxide nanoparticles. Biosynthesized CuO-NPs were characterized using various spectroscopic and analytical techniques and their biological potential was investigated using various *in vitro* assays. Moreover, the cytotoxicity of CuO-NPs was analyzed using NIH 3T3 fibroblast cell lines to predict the pharmacological application of CuO-NPs.

## 2. Materials and methods

### 2.1 Chemicals

Copper acetate monohydrate (molecular weight: 199.65 g mol^−1^) was purchased from Sigma Aldrich. Ethanol was purchased from Anal R NORMAPUR (UK). Trypsin-EDTA solution and Dulbecco's Modified Eagle Medium Low Glucose (DMEM-LG) was purchased from Sigma Aldrich USA. Fetal bovine serum (FBS) was acquired from Gibco. NIH 3T3 mouse embryonic fibroblast cell line was a gift from Interdisciplinary Research center in Biomedical materials (IRCBM), COMSATS University Islamabad, Lahore, Pakistan.

### 2.2 Seeds collection and preparation of seed extract

*Silybum marianum* seeds were obtained from Plant Cell and Tissue Culture Lab (Herbarium code: PSM 04), Department of Biotechnology, Quaid-i-Azam University Islamabad. Seeds were washed with distilled water twice, dried and grinded to form fine powder. 20 gram of seed powder was added to 200 mL of distilled water in a flask and stirred at 6000 rpm for 3 h at 50 °C to prepare aqueous extract. The extract was filtered to remove solid residues by passing through nylon cloth. The filtrate is further purified by passing through Whatman filter paper three times. The prepared extract was stored at 4 °C for further experiments.

### 2.3 CuO-NPs biosynthesis

Biosynthesis of CuO-NPs was conducted by adding copper acetate monohydrate to aqueous seed extract as described by ref. 39. Briefly, 10 gram of salt (copper acetate monohydrate) was dissolved in 100 mL of prepared extract and agitated for 2 h at 50 °C. After the completion of reaction the solution was centrifuged for 10 minutes at 10 000 rpm to settle down the dissolved precipitate and CuO-NPs in the form of pellet were collected. Pellet was washed thrice with distilled water and twice with ethanol *via* centrifugation for further purification and allowed to dry at 40 °C for 24 h. The obtained CuO-NPs were calcinated at 500 °C for 3 h to remove further impurities and get crystalline structure.

### 2.4 HPLC analysis

Phytochemicals conjugated to CuO-NPs were confirmed though high performance liquid chromatography (HPLC) consists of Varian Prostar 230 pump, Varian Prostar 335 Photodiode Array Detector (PAD), Metachem Degaist degasser, and Varian Prostar 410 autosampler, operated by Galaxie version 1.9.3.2 software (Varian, Le PlessisRobinson, France). During HPLC the standards for compound separation *i.e.* taxifolin, silychristin, isosilychristin, silydianin, silybin A, silybin B, isosilybin A, isosilybin B. were obtained from sigma Aldrich. For separation, Hypersil PEP 300 C18, 250 × 4.6 mm was used at 35 °C with a particle size of 5 μm prepared by guard column Alltech, 10 × 4.1 mm. Detection was made at wavelength of 320 nm and 520 nm. Mobile phase was composed of solvent A (HCOOH/H_2_O, pH = 2.1) and solvent B (CH_3_OH). The composition of mobile phase changes 1 h per run, with nonlinear gradient as follows: 8% B (36 min), 100% B (30–35 min), 33% B (28 min), 30% B (17 min), 12% B (11 min), and 8% B (0 min) at flow rate of 1 mL min^−1^. Among each run a 10 min re-equilibration time was used. Quantification was made on comparing with reliable reference standards and an assessment of retention time. Analysis was made three times for each sample. The results were measured as μg mg^−1^ DW of samples.

### 2.5 Physicochemical and morphological characterization of CuO-NPs

To determine structural, chemical and morphological characteristics of biosynthesized CuO-NPs various characterization techniques were carried out including XRD, FTIR, SEM and DLS. To confirm the synthesis of CuO-NPs, X-ray diffractometer was used. CuO-NPs were subjected to XRD instrument (Model-D8 Advance, Germany) in range of 10–70° with step time of 0.55 seconds. Data was analyzed through Cu Kα radiation with wavelength of 1.5406 Å. Debye–Scherrer equation was used to determine average size of CuO-NPs.
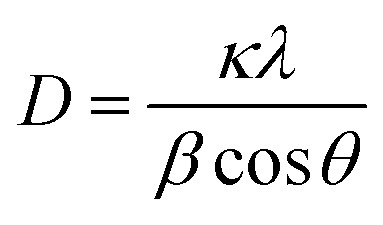
where *κ* = shape factor, *λ* = X-ray wavelength, *θ* = Bragg's angle, *β* = full length at half maximum in radians. Fourier Transform Infrared Radiation (FTIR) was performed with wavelength in range of 515 cm^−1^ to 4000 cm^−1^ for determination of attached functional groups that may act as reducing and capping agents. Morphological attributes were examined through scanning electron microscopy (SEM; JEOL, Tokyo, Japan), operated at 20 kV and 0.18 nm point to point resolution. Dynamic light scattering (DLS) of biosynthesized CuO-NPs was performed to determine surface charge and stability as well as to estimate size distribution of nanoparticles using Malvern zeta sizer.

### 2.6 Anti-diabetic potential of CuO-NPs

#### 2.6.1 α-Amylase inhibition assay

The anti-diabetic activity of synthesized CuO-NPs were analyzed by performing α-amylase inhibition assay described by ref. 40. Briefly, 15 μL of phosphate buffer (pH 6.8) and 25 μL of α-amylase enzyme (0.14 U mL^−1^) was added in micro wells of 96 wells plate, followed by addition of test sample and 40 μL of starch solution to the mixture. After 30 min of incubation at 50 °C, 20 μL of 1 M HCL was added to the wells followed by addition of 90 μL of iodine reagent (5 mM iodine, 5 mM potassium iodide). Negative control is represented by 100% enzymatic activity as it does not contain any test sample. Acarbose was considered as a positive control while solution without enzyme and test sample was considered as a blank.

Following formula is used to calculate percent enzyme inhibition:
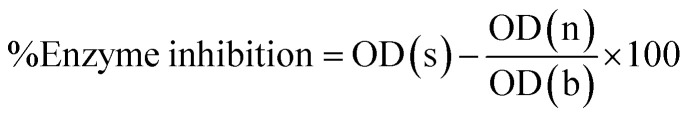
where OD (n) indicates negative control, OD (b) indicates blank, and OD (s) indicates absorbance value of test sample.

#### 2.6.2 Urease inhibition assay

To perform urease inhibition activity, 96 well plate containing reaction mixture comprised of 25 μL urease, 100 mM urea, 50 μL phosphate buffer (3 mM, pH adjusted to 4.5) and 10 μL of test sample was incubated at 30 °C. After 15 min incubation, 45 μL phenol reagent (phenol 1% (w/v) sodium nitroprusside 0.005% (w/v)) was added to the wells, followed by addition of 70 μL alkali reagent (0.1% NaOCl and 0.5% (w/v) NaOH). The reaction mixture was incubated at 30 °C for 50 minutes and %inhibition of urease was analyzed by recording absorbance at 630 nm using microplate reader as described by ref. 41. Thiourea was considered as positive control and mixture without test sample was used as a blank.

Enzyme inhibition was determined in percentage using following equation.
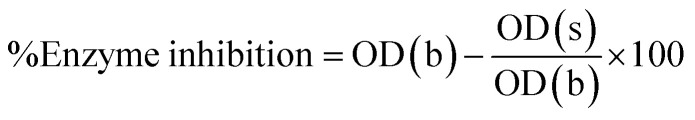
where OD (b) indicates blank, and OD (s) indicates value of sample.

#### 2.6.3 Lipase inhibition assay

Lipase inhibition activity was performed following previously described protocol with few modifications.^42^ Lipase enzyme (10 mg mL^−1^) was dissolved in water, centrifuged at 16 000 rpm for 5 minutes and supernatant was collected. The reaction mixture was prepared by adding 150 μL lipase, 350 μL buffer (Tris buffer (100 mM; pH 8.2)) and 50 μL of test sample into Eppendorf. To initiate the reaction 450 μL of substrate (olive oil) was added to each Eppendorf. Orlistat acts as an inhibitor while blank consists of 400 μL buffer, 150 μL lipase, and 450 μL substrate without test sample. The mixture was incubated at 37 °C for 2 h followed by centrifugation at 16 000 rpm. 200 μL supernatant was then poured to each well of microtiter plate and absorbance was recorded at 400 nm wavelength using a microplate reader.

Following formula expressed the percent inhibition of enzyme.
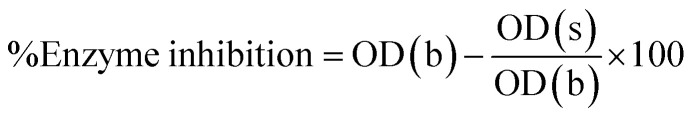
where OD (b) indicates blank, and OD (s) indicates value of sample.

### 2.7 Antioxidant activities of biosynthesized CuO-NPs

#### 2.7.1 Free radical scavenging assay (FRSA)

DPPH (2,2-diphenyl 1-picrylhydrazyl) assay was performed to analyze free radical scavenging potential of CuO-NPs using protocol reported by ref. 43. Briefly, 10 μL of sample and 190 μL of the DPPH reagent was added into 96 well plate and mixture was incubated for 30 min at 37 °C. Ascorbic acid act as a positive control. Using a microplate reader the absorbance of reaction mixture was measured at 515 nm. Following formula is used to calculate free radical scavenging potential:
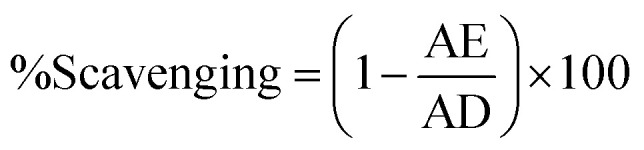


In above equation AE indicates absorbance of DPPH solution with sample and AD expresses negative control.

#### 2.7.2 Total antioxidant capacity (TAC)

Total antioxidant potential of CuO-NPs was evaluated using phosphomolybdenum based protocol previously described by ref. 44. Initially, 900 μL of phosphomolybdenum reagent (28 mM sodium phosphate, 0.6 M sulphuric acid and 4 mM ammonium molybdate) was mixed with ascorbic acid (positive control) and 100 μL of prepared test sample. After 90 min of incubation at 95 °C, 200 μL of the reaction mixture was poured in 96 well plate. The OD of the sample was obtained using microplate reader. The activity was measured as number of ascorbic acid equals to ascorbic acid per mg of test sample (μg AAE/mg), respectively.

#### 2.7.3 Total reducing power (TRP)

Potassium ferricyanide based reducing power assay for biosynthesized CuO-NPs was performed by protocol described by ref. 44. Iron was used as a reducing agent to investigate total reducing power of CuO-NPs. 40 μL of test sample was added into Eppendorf tubes followed by addition of 400 μL phosphate buffer (0.2 mol l^−1^, pH 6.6) and potassium ferricyanide (1% w/v in H_2_O). The Eppendorfs containing the reaction mixture were kept at 45 °C for 20 min followed by addition of 400 μL trichloroacetic acid (10% w/v in H_2_O). After centrifugation for 10 min at 3000 rpm the supernatant was collected. 500 μL of the collected supernatant was mixed with equal amount of distilled water and 100 μL of FeCl_3_ (0.1% w/v in H_2_O) in 96 well plate. Absorbance was measured at 630 nm using spectrophotometer and the calculation was made as μg AAE/mg. DMSO act as blank while ascorbic acid served as a positive control.

#### 2.7.4 ABTS antioxidant assay

ABTS (2,2-azinobis-3-ethylbenzothiazoline-6-sulphonic acid) assay was performed by mixing potassium per sulphate, 2.5 mM with equal proportion of 7 mM ABTS salt. The solution was kept in dark for 16 h as described by ref. 45. Before adding CuO-NPs to the reaction mixture, absorbance was recorded at 734 nm and was adjusted to 0.7. CuO-NPs were mixed with the reaction mixture and kept in dark for 15 min at room temperature (25 ± 1 °C). Absorbance was recorded at 734 nm and the activity was expressed in TEAC (trolox C equivalent antioxidant capacity, μM).

#### 2.7.5 FRAP (ferric reducing antioxidant power) assay

Ferric reducing antioxidant power of CuO-NPs was evaluated using protocol mentioned by ref. 46. For this purpose, 10 μL of the test sample was added to 190 μL of FRAP solution [2,4,6-tri(2-pyridyl)-*s*-triazine (TPTZ; 10 mM); ferric chloride hexahydrate (FeCL_3_·6H_2_O) 20 mM, acetate buffer (300 mM) of pH 3.6; ratio 10 : 1 : 1 (v/v/v)]. The reaction mixture was kept at room temperature for 15 min and absorbance was taken at 630 nm. The activity was determined as TEAC and the assay was conducted in triplicates.

### 2.8 Antibacterial activity

Antibacterial potential of CuO-NPs was investigated using five different pathogenic bacterial strains including *Salmonella typhi*, *Enterobacter aerogenes*, *Salmonella setubal*, *Micrococcus luteus* and *Staphylococcus aureus* using disc diffusion method.^40^ Bacterial culture was adjusted to required maximum optical density (OD) of 0.5 by adding sterilized culture broth in a proper ratio. The bacterial strains were then lawn evenly on the plates using autoclaved cotton buds. Sterile discs of filter paper loaded with 5 μL (4 mg mL^−1^ and 20 mg mL^−1^ CuO-NPs in DMSO) of samples were applied on the inoculated media and the culture was allowed to incubate for 24 h at 37 °C. Cefexime served as positive control. The appeared zone of inhibitions were measured around samples and control and zone of inhibition ≥12 mm was considered as significant.

### 2.9 Cell viability assay (XTT assay)

Cytotoxicity of biosynthesized CuO-NPs was assessed by XTT assay (2,3-bis[2-methoxy-4-nitro-5-sulfoxyphenyl]-2*H*-tetrazolium 5-carboxyanilide inner salt), against NIH 3T3 fibroblast cells using XTT assay kit (Roche, Switzerland) as described by ref. 47. Cultured fibroblast cells when reached at 85% confluency were trypsinized (using 1X trypsin-EDTA solution) and were seeded in 96 well plate (3000 cells per well) providing the complete growth medium, Dulbecco's Modified Eagle Medium Low Glucose (DMEM-LG) supplemented with 20% fetal bovine serum (FBS). The plate was incubated at 37 °C in the 5% CO_2_ incubator for 24 h. After overnight incubation the cells were treated with 20 μg mL^−1^, 50 μg mL^−1^, 75 μg mL^−1^ and 100 μg mL^−1^ concentration of CuO-NPs suspended in serum free low LG medium for 24 h. The group including cells seeded on simple tissue culture plate without any treatment of CuO-NPs is considered as control. After 24 h treatment, the wells media was aspirated and washed twice with 1XPBS. 50 μL of fresh prepared mixture of XTT (with electron coupling reagents prepared in ratio of 50 : 1) was added to corresponding wells of plate. The plate was then wrapped completely in aluminum foil and kept in 5% CO_2_ incubator at 37 °C. The absorbance was recorded at wavelength of 450 nm with 630 nm as a reference wavelength. The experiment was repeated twice in a triplicate manner.

### 2.10 Measurement of peroxidase-like activity of CuO-NPs

Peroxidase (POD) activity of synthesized CuO-NPs was evaluated by protocol described by ref. 48. Briefly, each well of microtiter plate was filled with 140 μL NaAc–HAc buffer (0.2 M, pH 4.0) and a 20 μL test sample was loaded followed by addition of H_2_O_2_ (6 mM, freshly prepared) and 20 μL of TMB (3 mM, freshly prepared). Reaction mixture without sample was considered as a control and absorbance was taken at 652 nm wavelength using spectrophotometer. Enzymatic activity was determined by applying following formula:*A* = *ELC*

In above equation *A* represents value of sample absorbance, *C* represents enzyme concentration (mM min^−1^ mg^−1^), *E* is extinction coefficient and *L* indicates length of wall, respectively.

### 2.11 Detection of reactive oxygen/nitrogen species

To determine the level of ROS and RNS generated by application of CuO-NPs, dihydrorhodamine-123 (DHR-123) fluorescent dye was used.^46^ For this purpose, yeast cells were incubated for overnight in presence of CuO-NPs and DMSO (control cells). Cells were placed in the dark at 30 °C for 10 min after washing with PBS twice and resuspended in PBS with 0.4 μM DHR-123. BioRad VersaFluor Fluorimeter (*λ*_ex_ = 505 nm, *λ*_em_ = 535 nm) was used to detect fluorescence after washing with PBS.

### 2.12 Statistical analysis

The experiments were performed in triplicate and the data was evaluated to find out mean values and standard deviation (means ± SD) by using SPSS (Windows version 7.5.1, SPSS Inc., Chicago, IL, USA). One-way ANOVA (analysis of variance) accompanied by unpaired Bonferroni test was used to analyze results of cell viability assay, ROS/RNS activity, peroxidase like activity and lipase, urease and amylase inhibition assays. *P* value <0.05 indicates that results are statistically significant.

## 3. Results and discussion

### 3.1 Characterization of biosynthesized CuO-NPs

#### 3.1.1 Synthesis and HPLC analysis of CuO-NPs

**Fig. 2:**
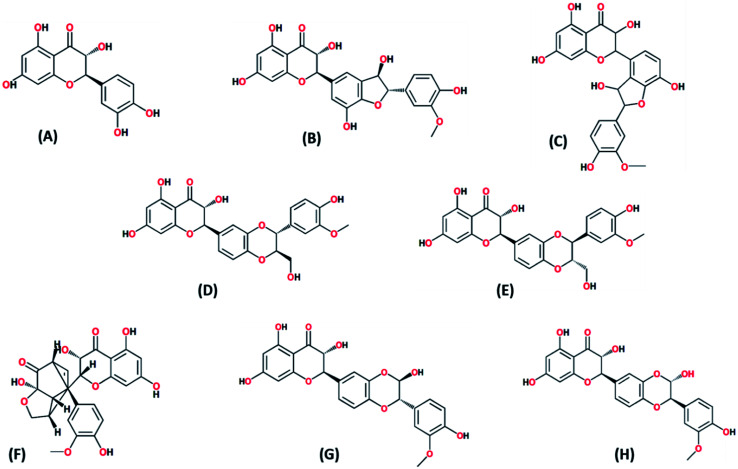
Bioactive silymarin compounds identified by HPLC as capping agent on CuO-NPs. (A) Taxifolin; (B) silychristin; (C) isosilychristin; (D) silybin A; (E) silybin B; (F) silydianin; (G) isosilybin A; (H) isosilybin B.

In the present study, *Silybum marianum* seeds extract was exploited for biosynthesis of CuO-NPs. Fine blackish powder of CuO-NPs was obtained after washing, drying, and annealing and stored in tight capped glass vial at 4 °C, until further process for morphological, physiochemical and biological activities. Quantification of silymarin compounds were obtained by HPLC of CuO-NPs as described in previous articles.^49^ Silymarin compounds (Fig. 2) were analyzed, and their presence indicates that these phytochemicals might act as capping and stabilizing agents in the biosynthesis of CuO-NPs. Results showed that silybin A was present in maximum quantity while taxifolin was the least one with total silymarin content of 2.55 ± 0.08 mg g^−1^ DW (Table 1). These phytochemicals owing to their reduction capacities might have served as a reducing agent for reduction of precursor salt into CuO-NPs. Previously, it has also been reported that silymarin has a strong reducing potential.^33,50^ Our results are in harmony and supported by previous report.^51^

**Table 1:** Quantification of bioactive phytochemical compounds by HPLC in biosynthesized CuO-NPs

Phytochemical compounds								
Compounds	Taxifolin	Sily-christin	Iso-silychristin	Silydianin	Silybin A	Silybin B	Iso silybin A	Iso-silybin B
Conc. (μg g^−1^ DW)	3.70 ± 1.11	135.53 ± 1.62	266.81 ± 1.33	686.31 ± 1.64	705.06 ± 1.59	451.15 ± 1.76	161.83 ± 1.47	136.80 ± 1.52
Total silymarin (mg g^−1^ DW)	2.55 ± 0.08							

#### 3.1.2 X-ray Diffraction (XRD) analysis

**Fig. 3:**
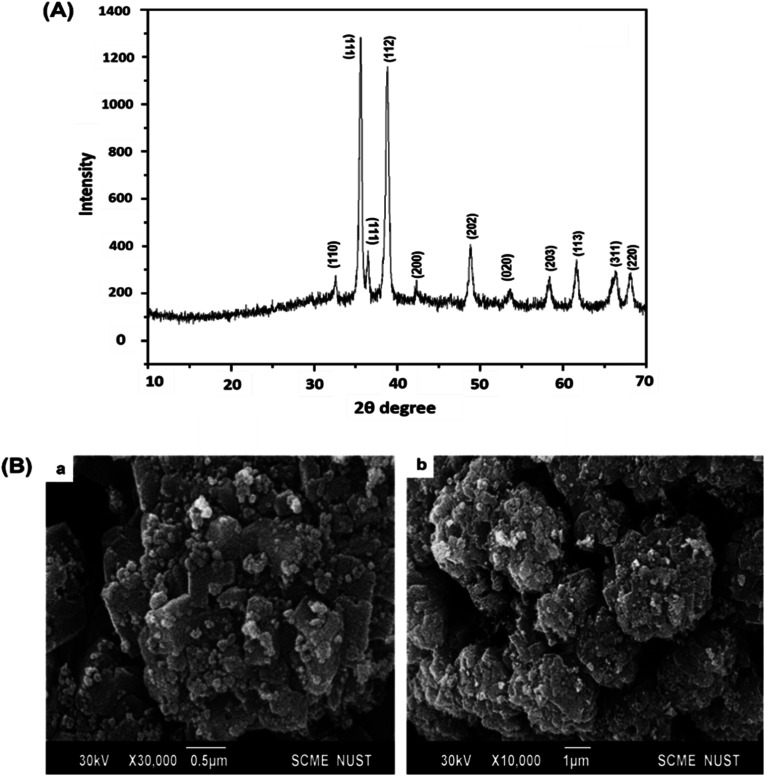
XRD Pattern (A) and SEM micrographs (B) of *Silybum marianum* mediated CuO-NPs.

X-ray diffraction pattern of biosynthesized CuO-NPs was obtained with diffraction angles from 10 to 70, indicating purity and structural morphology of CuO-NPs. Strong peaks were observed at 32.64°, 35.56°, 36.03°, 38.78°, 43.04°, 48.84°, 53.71°, 58.31°, 61.62°, 66.41° and 68.07° (Fig. 3A). Miller indices of the CuO-NPs were (110), (111), (111), (112), (200), (202), (020), (203), (113), (311) and (220), confirmed with JCPDS file no. 048-1548.^52^ Data indicates crystalline monoclinic phase with average size of 15 nm using the Debye Scherrer equation. These results are supported by the previous reports.^53,54^

#### 3.1.3 Scanning Electron Microscopy (SEM)

Morphological characteristics of biosynthesized CuO-NPs were investigated by performing SEM. SEM micrograph shows surface morphology of CuO-NPs exhibiting spherical shape with some extent of agglomeration (Fig. 3B). Aggregation of particles in the form of connected spherical structure were observed. Similar results were reported by previous studies.^55–57^

#### 3.1.4 FTIR

**Fig. 4:**
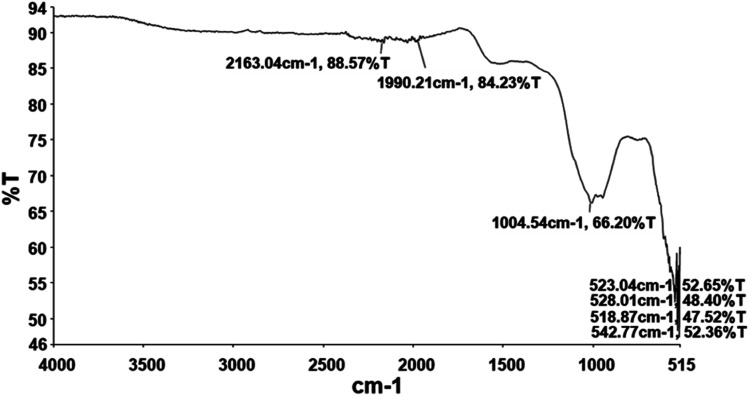
FTIR spectra of biosynthesized CuO-NPs.

Fourier Transform Infrared Spectroscopy (FTIR) identifies attached functional groups by measuring vibrational frequencies of bond in the molecule. Fig. 4 indicates the FTIR spectra of biosynthesized Cu-NPs exhibiting characteristics peaks at 2163 cm^−1^ and 1990 cm^−1^ which corresponds to S–C≡N and N=C=S stretch of thiocyanate and isothiocyanate groups. The peak at 1004 cm^−1^ may corresponds to C–O (C–O–C) stretch indicating ether, pyranose ring and glycosidic linkage.^58^ The peaks at the fingerprint regions are at 518, 523, 528 and 542 indicating Cu–O stretching. These peaks confirms the formation of CuO nanostructure as reported previously.^59,60^

#### 3.1.5 Dynamic light scattering

**Fig. 5:**
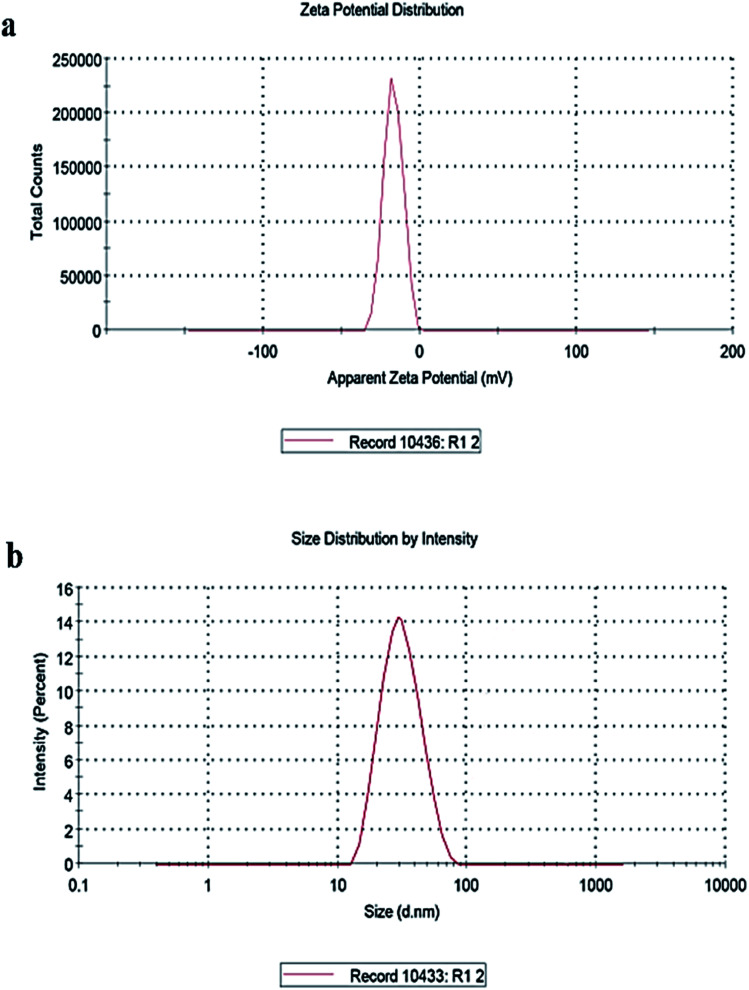
(a) Zeta potential showing the net charge and stability of CuO-NPs, (b) zeta sizer indicating the average size of CuO-NPs.

CuO-NPs were analyzed to determine their colloidal stability, surface charge and size distribution by performing zeta potential and zeta sizer using dynamic light scattering. Zeta potential determines the net charge of −16.9 mV on CuO-NPs (Fig. 5a) conferring more colloidal stability to the CuO-NPs. The negative charge on CuO-NPs may be due to binding of phytochemical compounds to the CuO-NPs. Zeta sizer measures the size distribution of nanomaterial and the results showed that 35.5 nm is the average size of synthesized CuO-NPs with poly disperse index (Pdi) of 0.136 (Fig. 5b). These results are in accordance with the prior studies.^61^

### 3.2 Anti-diabetic potential of CuO-NPs

#### 3.2.1 α-Amylase inhibition assay

**Fig. 6:**
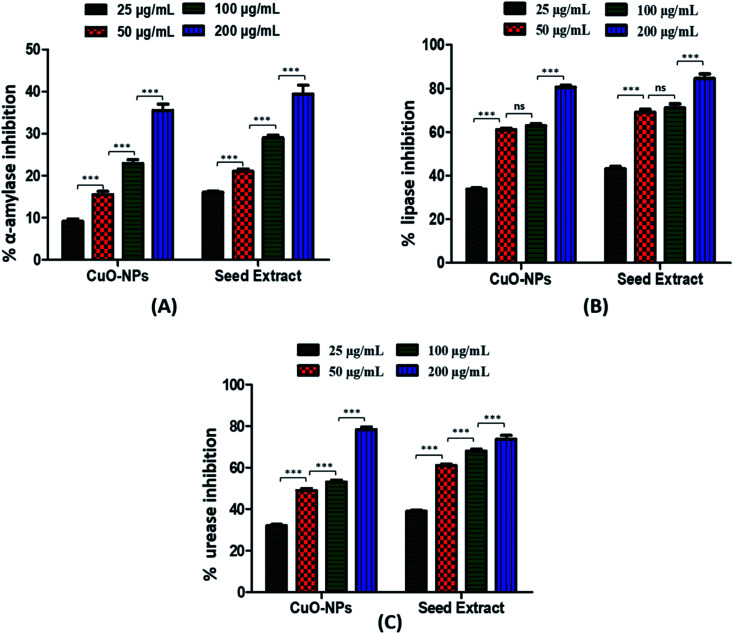
Graphical representation of α-amylase (A), lipase (B) and urease (C) inhibitory potential of CuO-NPs.

One of the serious and prevalent cause of morbidity and mortality around the globe is diabetes and its related complications. *Diabetes melitus* (DM) a metabolic disorder, is characterized by chronic hyperglycemia induced by a reduction in insulin synthesis or body cells insensitivity to insulin.^62^ Apart from anti-diabetic drugs that are used to control post-prandial hyperglycaemia, some plant-based compounds have been found useful as inhibitors to reduce starch hydrolysis and hence, are considered an attractive candidate to treat diabetes mellitus. Paving a role in this research, the *Silybum marianum* seed extract mediated CuO-NPs were exposed to α-amylase inhibition assay to determine enzyme inhibition potential of synthesized particles. Results showed that synthesized CuO-NPs exhibited moderate level of inhibition for the α-amylase enzymes which is comparable to the percent inhibition caused by extract. CuO-NPs caused 35.5 ± 1.54% of inhibition of α-amylase enzyme at 200 μg mL^−1^ (Fig. 6A). Our findings proposed that the CuO-NPs of *Silybum marianum* seed extract have considerable enzyme inhibitory potential. The α-amylase inhibitory potential of medicinal plants can be attributed to numerous possible factors, including fibre concentration, encapsulation of enzyme and starch by the fibres when present in reaction mixture and the inhibitors on the fibres surface, thus causing direct adsorption of the α-amylase enzyme on the fibres surface, and reducing availability of starch to the enzyme, results in decreased α-amylase activity.^63^ Previous reports showed α-amylase inhibition potential of CuO-NPs and the inhibition pattern is relatable to our findings.^26,64^

#### 3.2.2 Urease inhibition assay

Urease is a biological active enzyme that hydrolyses urea in to carbon dioxide and ammonia. Many microorganisms metabolizes the urea through enzymatic action of urease that's why urea occur abundantly in biologically active soil.^65^ Results from urease inhibitory assay performed using biosynthesized CuO-NPs revealed that CuO-NPs exhibits excellent urease inhibitory potential by showing percent inhibition of 78.4 ± 1.26% when compared to the thiourea (standard urease inhibitor) at 200 μg mL^−1^ (Fig. 6C). This inhibitory effect might be attributed to the functional groups that bind to the CuO-NPs during biosynthesis process.

#### 3.2.3 Lipase inhibition assay

Lipases are the group of enzymes that catalyzes the hydrolysis of triglycerides in to fatty acid and glycerol molecule. These fat splitting enzymes are present in blood, gastric juices and pancreatic secretions *etc.*^66^ Results from lipase inhibitory assay performed using biosynthesized CuO-NPs revealed that CuO-NPs exhibits high inhibition potential for the lipases. CuO-NPs showed percent inhibition of 80.5 ± 0.91% at 200 μg mL^−1^ which is comparable to the inhibition potential of extract alone (Fig. 6B). The inhibitory effect can be due to the phytochemicals conjugated to CuO-NPs during biosynthesis process. The presence of phytochemicals and functional groups such as hydroxyl group (–OH) and carbonyl group (C=O) may be responsible for this inhibitory property.^65,67^

### 3.3 Antioxidant potential of CuO-NPs

**Fig. 7:**
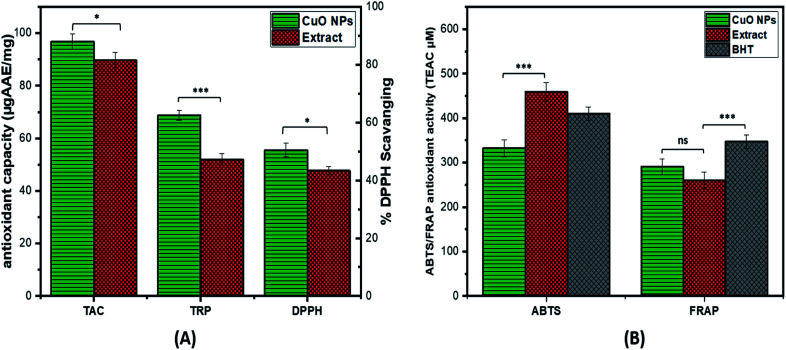
Graphical representation of antioxidant activities of CuO-NPs: (A) shows total antioxidant capacity (TAC), total reducing potential (TRP) and %DPPH scavenging activity while (B) shows ABTS and FRAP antioxidant activities.

The antioxidant potential of CuO-NPs is investigated by performing TAC, TRP, DPPH, ABTS and FRAP assay (ET based antioxidant activity). The value of TAC was found to be high *i.e.* 96.9 ± 0.26 μg AAE/mg for CuO-NPs as compared to the extract. The TRP was performed to further assess the antioxidant potential of synthesized CuO-NPs. The reducing potential of CuO-NPs is investigated by assessing the presence of reductones species that are reducing agents and believed to have H-atom donating capacity. The TRP value of CuO-NPs is 68.8 ± 0.35 μg AAE/mg which is higher as compared to plant extract. Furthermore, DPPH assay was carried out in which DPPH moiety accepts electrons from the donor species and get reduced resulting in creation of light-yellow diphenyl picrylhydrazine molecule. At 4 mg mL^−1^ CuO-NPs concentration high DPPH radical scavenging was observed *i.e.* 55.5 ± 0.62% as compared to the extract alone (Fig. 7A). The ABTS value for the synthesized CuO-NPs was high *i.e.* 341.79 μM expressed as TEAC, similarly FRAP results also showed high value for the synthesized CuO-NPs 269.43 μM (Fig. 7B). From the above results it can be assumed that some of the compounds with antioxidant capacity from *Silybum marianum* seed extract might have involved in reducing and stabilizing the CuO-NPs during the biosynthesis, thus enhancing the antioxidant capacity of synthesized CuO-NPs. These results of antioxidant activities are supported by the previous results.^51,68^

### 3.4 Antibacterial assay

**Fig. 8:**
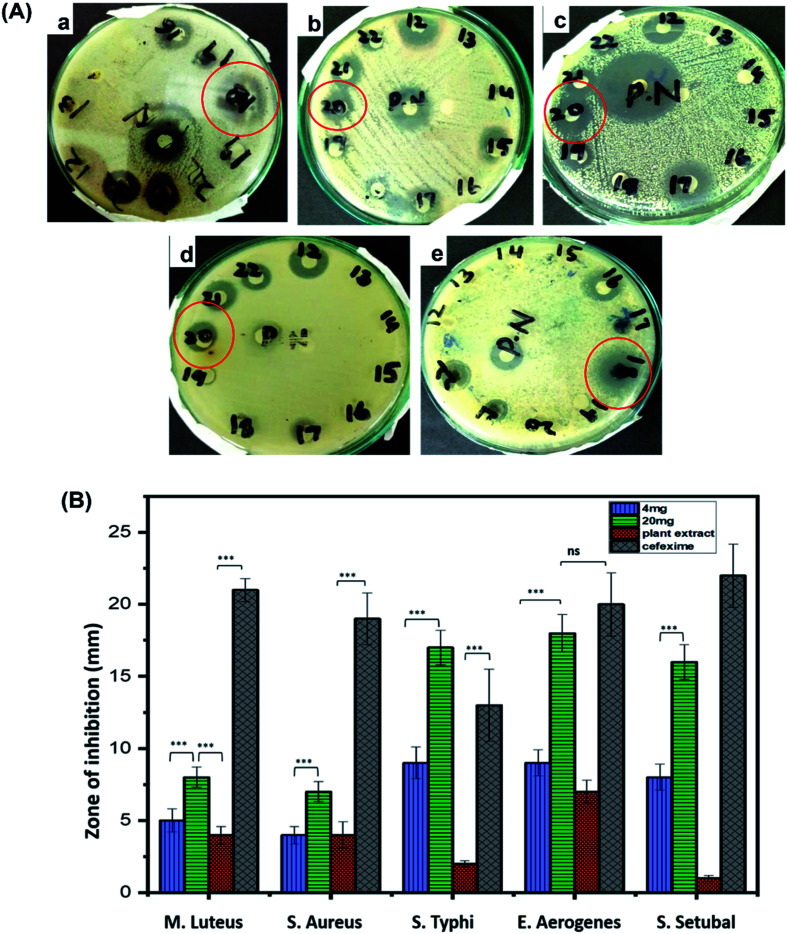
Figure (A) exhibits pictorial illustration of antibacterial activity of CuO-NPs against *Klebsiella aerogenes* (a), *Micrococcus luteus* (b), *Salmonella setubal* (c), *Staphylococcus aureus* (d) and *Salmonella typhi* (e) and (B) shows graphical representation of antibacterial activity of CuO-NPs against respective strains.

Antibacterial potential of biosynthesized CuO-NPs was investigated against five bacterial strains, using disc diffusion method. Overall, CuO-NPs exhibited efficient antibacterial activity against all tested strains but *Enterobacter aerogenes* and *Salmonella typhi* were the most susceptible. Clear zone of inhibitions for these two strains were observed in 24 h incubated cultures supplemented with CuO-NPs loaded discs. The measurement was made in millimeter using vernier caliper calliper. Zone of inhibitations at 4 mg mL^−1^ were recorded as 5 ± 0.8 mm for *Micrococcus luteus*, 4 ± 0.6 mm for *Staphylococcus aureus*, 9 ± 1.1 mm for *Salmonella typhi*, 9 ± 0.9 mm for *Enterobacter aerogenes* and 8 ± 0.9 for *Salmonella setubal*, similarly at 20 mg mL^−1^ of concentration zone of inhibitations were observed as 8 ± 0.7 mm for *Micrococcus luteus*, 7 ± 0.7 mm for *Staphylococcus aureus*, 17 ± 1.2 mm for *Salmonella typhi*, 18 ± 1.3 mm for *Enterobacter aerogenes* and 16 ± 1.2 mm for *Salmonella setubal*, respectively (Fig. 8). At higher concentration of CuO-NPs (20 mg mL^−1^) applied, the greater bactericidal effect of CuO-NPs was observed compared to 4 mg mL^−1^ of concentration. The reason for growth inhibition can be the possible interaction between external membrane of bacteria and the CuO-NPs. CuO-NPs may disrupt the integrity of cell membrane causing malfunctioning of enzymes and increasing cell permeability leading to bacterial cell death.^25,69^ Moreover, CuO-NPs may integrate inside the cell membrane due to smaller size than pores on bacterial cell membrane. CuO-NPs also produce superoxide and hydroxyl free radicals (reactive oxygen species) and damage the cell by oxidizing double bonds in phospholipids and disrupt the cell permeability which results in high osmotic stress and ultimately cause death of bacterial cell.^70,71^

### 3.5 Cytotoxicity of CuO-NPs against NIH3T3 cells

**Fig. 9:**
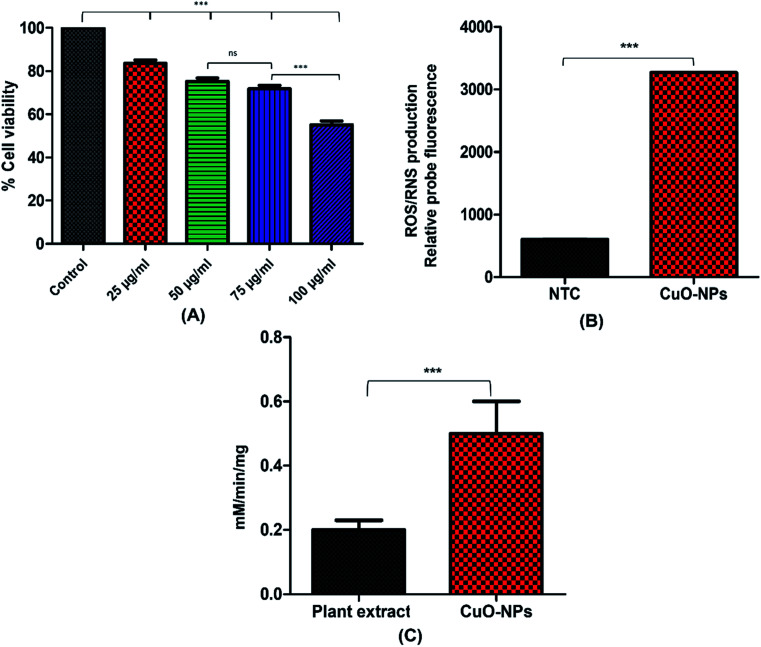
Figure (A) shows graphical representation of cytotoxicity of CuO-NPs against NIH3T3 fibroblast cells, (B) ROS/RNS measurement against yeast cells and (C) shows peroxidase-like catalytic activity of CuO-NPs.

NIH3T3 fibroblast cell lines were used to examine the cytotoxic effect of CuO-NPs. Results showed dose dependent cytotoxicity of CuO-NPs, indicating that with the increase in the concentration of CuO-NPs, the percent cell viability decreases. The XTT assay results indicated %viability (100.0 ± 0.022% in control group *vs.* 83.60 ± 1.505% in 25 μg mL^−1^ group, 75.3 ± 1.49% in 50 μg mL^−1^ group, 71.8 ± 1.59% in 75 μg mL^−1^ and 55.1 ± 1.80% in 100 μg mL^−1^) as shown in Fig. 9A. The results showed that 25 μg mL^−1^ concentration of CuO-NPs offer little toxicity to the cells as compared to control while with the increase in CuO-NPs dose, the cell viability gradually decreases indicating that 50 μg mL^−1^ and 75 μg mL^−1^ are slightly more toxic to the cells compared to control and 25 μg mL^−1^ dose. At 100 μg mL^−1^ concentration the cell viability becomes reduced to half exhibiting that this dose of CuO-NPs is toxic to fibroblast cells. These results indicated that 100 μg mL^−1^ and above doses of biosynthesized CuO-NPs can be lethal to the fibroblast cells. With the increase in the use of metallic nanoparticles for biomedical applications, the nanotoxicity associated with nanoparticles is also becoming matter of concern. Over production and extended use of nanoparticles causes serious issues to the human health.^72^ Similar pattern for cytotoxicity was observed in the previous report,^73^ however the percent cell viability was higher in previous report, the possible reason for reduced cell viability in the present study might be due to the small size of synthesized CuO-NPs which may result in their more easy penetration inside the cells, causing higher rate of cell death. In case of more nanoparticles penetration inside cells, enzymes leaks out of the cell as the cell membrane integrity damages causing cell death. When cells are treated with high level of CuO-NPs the level of ROS also increase resulting in elevated oxidative stress. The oxidative stress is mainly induced in the mitochondria that leads to initiation of apoptosis inside the cell.^73^

### 3.6 Peroxidase-like activity of CuO-NPs

The ability of green CuO-NPs to break down hydrogen peroxide (H_2_O_2_) was determined by performing peroxidase (POD) activity assay. Peroxidases are abundantly present in plants and animals. These enzymes catalyzes the breakdown of H_2_O_2_, causing oxidation of many phenolic and non-phenolic compounds. Results showed that the biosynthesized CuO-NPs exhibited lesser catalytic activity as compared to the extract (Fig. 9C). The catalytic activity of CuO-NPs was 0.5 mM min^−1^ mg^−1^ while the seed extract showed higher activity of 1.5 mM min^−1^ mg^−1^, respectively. Our results are in accordance with the previous study which demonstrated the peroxidase-like catalytic activity of CuO-NPs through formation of blue color product after addition of CuO-NPs to the medium containing TMB as peroxidase substrate.^74^ This catalytic property of CuO-NPs makes them an ideal candidate as peroxidase mimics for a number of potential applications.

### 3.7 Reactive oxygen and nitrogen species measurement

ROS and RNS are generated as a by-product of metabolism in mitochondria. To evaluate the level of ROS/RNS dihydrorhodamine 123 (DHR 123) probe was used. The results shown in Fig. 9B indicated that CuO-NPs increases the production of ROS/RNS within yeast cells as compared to the NTCs (control). Incubation of CuO-NPs with yeast cells cases the generation of ROS/RNS up to 3265 whereas ROS/RNS production in NTCs was 602 in contrast to the CuO-NPs. Mitochondrial respiration generally generates the free radical species specifically electron transport chain is the site where oxygen leakage and production of ROS occur. Application of metallic nanoparticles enhanced the production of free radicals by Fenton reaction. Also metallic ion of nanoparticles can inhibit electron transport at mitochondria resulting in increased production of ROS. Similar results were reported previously, where application of metallic nanoparticles induced the production of ROS.^75^ Increase in ROS/RNS causes damage to the cell due to imbalance between free radical species and their biological scavenging activity.

## 4. Conclusion

In current study, CuO-NPs were synthesized *via* green approach: using seed extract of *Silybum marianum*. Biosynthesis of CuO-NPs is more environmental friendly, nontoxic, cheap, and easy. The biosynthesized CuO-NPs exhibited well defined physiochemical characteristics, spherical morphology and small size of 15 nm. Moreover, the biosynthesized CuO-NPs revealed broad spectrum antibacterial potency against both Gram positive and Gram-negative bacterial strains used in study, but *Enterobacter aerogenes* and *Salmonella typhi* were the most susceptible against CuO-NPs. The effective antimicrobial potential of CuO-NPs suggested their potential use as a precursor novel drug candidate against human pathogenic bacterial strains. CuO-NPs also revealed promising enzymatic inhibitory potential against lipases, ureases and alpha amylase and can be used in anti-diabetic based therapies. In addition, CuO-NPs displayed efficient peroxidase-like catalytic activity and ROS/RNS inhibition potential. Furthermore, CuO-NPs showed significant antioxidant potential and dose dependent cytotoxic effect against fibroblast cells. These results indicated that these biosynthesized CuO-NPs could be a potential candidate for biomedical applications.

## Funding

No fund was taken from any source.

## Conflicts of interest

All the authors declared no competing interest.

## Author contributions

JI and BHA conceptualized the research work. JI, SD, BM and HA performed experiments. GZ assisted in research work and HF and HJ provide reagents. AM and AA performed cell viability assay. JI wrote the manuscript and CH, MN and NG helped in writing and editing manuscript. AA assisted in data analysis and figure preparation. BHA supervised the work.

## Supplementary Material
